# Acetaminophen-induced anion gap metabolic acidosis secondary to 5-oxoproline: a case report

**DOI:** 10.1186/1752-1947-8-409

**Published:** 2014-12-06

**Authors:** Tarig Mohammed Abkur, Waleed Mohammed, Mohamed Ali, Liam Casserly

**Affiliations:** Department of Nephrology, University Hospital Limerick, Limerick, Ireland

**Keywords:** 5-oxoproline, Acetaminophen, High anion gap metabolic acidosis

## Abstract

**Introduction:**

5-oxoproline (pyroglutamic acid), an organic acid intermediate of the gamma-glutamyl cycle, is a rare cause of high anion gap metabolic acidosis. Acetaminophen and several other drugs have been implicated in the development of transient 5-oxoprolinemia in adults. We believe that reporting all cases of 5-oxoprolinemia will contribute to a better understanding of this disease. Here, we report the case of a patient who developed transient 5-oxoprolinemia following therapeutic acetaminophen use.

**Case presentation:**

A 75-year-old Caucasian woman was initially admitted for treatment of an infected hip prosthesis and subsequently developed transient high anion gap metabolic acidosis. Our patient received 40g of acetaminophen over a 10-day period. After the more common causes of high anion gap metabolic acidosis were excluded, a urinary organic acid screen revealed a markedly increased level of 5-oxoproline. The acidosis resolved completely after discontinuation of the acetaminophen.

**Conclusion:**

5-oxoproline acidosis is an uncommon cause of high anion gap metabolic acidosis; however, it is likely that it is under-diagnosed as awareness of the condition remains low and testing can only be performed at specialized laboratories. The diagnosis should be suspected in cases of anion gap metabolic acidosis, particularly in patients with recent acetaminophen use in combination with sepsis, malnutrition, liver disease, pregnancy or renal failure. This case has particular interest in medicine, especially for the specialties of nephrology and orthopedics. We hope that it will add more information to the literature about this rare condition.

## Introduction

Transient 5-oxoprolinemia is a rare clinical condition caused by glutathione deficiency. It usually presents as severe anion gap metabolic acidosis. The clinical manifestations usually reflect the acidosis and include altered mental state and hyperventilation. Several drugs have been implicated in the development of this disorder in patients who are predisposed. The diagnosis can be confirmed by testing the urine for organic acids. Discontinuation of the responsible medication leads to complete clinical and biochemical recovery.

## Case presentation

A 75-year-old Caucasian woman underwent a dynamic hip screw operation for treatment of an intertrochanteric fracture of her left hip. Six weeks later, she experienced a peri-prosthetic fracture, which was managed by hemiathroplasty. She was discharged to a rehabilitation center but readmitted four weeks later with suspected prosthesis infection.

Blood tests on admission showed neutrophil leukocytosis at 18.5×10^9^/L, elevated C-reactive protein at 210mg/L and bicarbonate of 24 mmol/L. Our patient underwent a hip revision.

Intra-operative specimens and blood cultures identified coagulase-negative *Staphylococcus aureus* that was sensitive to vancomycin. She started vancomycin (1g intravenously twice daily), along with acetaminophen (1g every six hours) for pain control. Her other blood work, including liver and kidney function tests, matched her baseline results, with normal liver function test results and a serum creatinine of 152μmol/L.

Her past medical history included chronic kidney disease with a baseline creatinine level between 130 and 150μmol/L, hypertension, non-insulin-dependent diabetes mellitus, congestive heart failure, dyslipidemia, chronic obstructive pulmonary disease, and folate deficiency. Her prescription medications included aspirin 75mg, bisoprolol 10mg, atorvastatin 10mg, furosemide 40mg twice daily, gliclazide modified release 90mg, pantoprazole 40mg, folic acid 5mg, combivent nebulizer, and OxyNorm® (oxycodone) 10mg as needed.

On day 10 of admission, our patient’s condition deteriorated and she became drowsy, confused and disorientated, and was hyperventilating. A physical examination revealed a Glasgow Coma Scale score of 12. Her vital signs were as follows: temperature, 36.2°C; blood pressure, 145/66mmHg; pulse, 80 beats per minute; respiratory rate, 24 breaths per minute; and oxygen saturation (SaO_2_), 99% on room air. Our patient moved all of her extremities spontaneously. The rest of her physical examination was unremarkable.

An electrocardiogram showed sinus rhythm of 90 beats per minute. A chest X-ray revealed mild cardiomegaly with no signs of infection or congestion. Repeat blood tests showed the following: sodium, 142mmol/L; potassium, 3.5mmol/L; chloride, 118mmol/L; CO_2_, 5mmol/L; urea, 8mmol/L; and creatinine, 150μmol/L. Liver function findings were unremarkable except for hypoalbuminemia at 17g/L. Full blood count findings showed hemoglobin of 10g/dL, neutrophil leukocytosis at 16.5×10^9^/L and platelets of 485×10^9^/L. Arterial blood gas showed metabolic acidosis with respiratory compensation (pH, 7.18; partial pressure of carbon dioxide, 1.7kPa; partial pressure of oxygen, 16.7kPa; bicarbonate, 8.3mmol/L; base excess, −22.7mmol/L; SaO2, 98.6% in room air). The corrected anion gap for hypoalbuminemia was 25mmol/L. The following causes of high anion gap metabolic acidosis (HAGMA) were excluded: lactic acid 0.7mmol/L, blood ketones <0.2mmol/L, salicylate <0.4mmol/L.

It was felt that her unchanged creatinine of 150μmol/L was insufficient to explain the marked deterioration in her acid–base status. A urine analysis and serum toxicology screen were unremarkable, including her serum acetaminophen level. The osmolar gap was mildly elevated at 21 mOsm/kg. Because her serum toxicology screen was negative, our patient’s urine was sent for organic acid detection by gas chromatography–mass spectrometry, which demonstrated a markedly increased excretion of 5-oxoproline at the peak of her acidosis, 10 days into her admission.

The acute management (based on the differential diagnosis and our patient’s condition) included commencement on bicarbonate (8.4% infusion of which she received a total of 600 milliequivalents over 48 hours), supportive intravenous fluids, and discontinuation of acetaminophen.

Over the following two days, her acidosis resolved with an overall improvement in her clinical condition. Repeat arterial blood gas showed the following: pH, 7.40; partial pressure of carbon dioxide, 3.8 kPa; partial pressure of oxygen, 12.7kPa; and bicarbonate, 20mmol/L. The acidosis correction persisted on subsequent testing over the remainder of her admission, indicating that the causative agent (acetaminophen) had been removed. The diagnosis of 5-oxoprolinemia was confirmed on receipt of organic acid test results two weeks later.

## Discussion

Elevated levels of plasma lactate, ketones and uremia are common causes of HAGMA. A less frequent cause is the temporary accumulation of the organic acid 5-oxoproline [1, 2]. 5-oxoproline is primarily metabolized to glutamate by the enzyme 5-oxoprolinase and is eliminated by the renal system.

**Figure 1:**
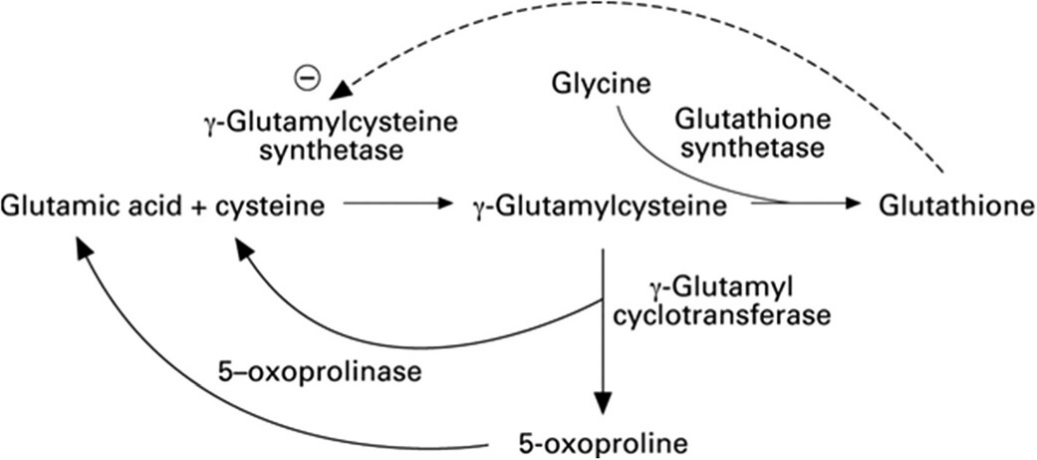
**Gamma-glutamyl cycle.** This figure shows the various compounds, enzymes and coenzymes that are required to form glutathione.

5-oxoprolinemia is classically caused by lack of glutathione [3]. 5-oxoproline is an intermediate in the gamma-glutamyl pathway, which is the metabolic cycle responsible for generating glutathione and membrane transport of amino acids into the cytosol [4]. In this pathway, normal glutathione stores are required for feedback suppression of the enzyme gamma-glutamylcysteine synthetase, which controls the activity of the cycle (see Figure 1).

The absence of glycine or reduced activity of the enzyme glutathione synthetase leads to accumulation of gamma-glutamylcysteine and its conversion to 5-oxoproline through an alternative pathway.

There is some polymorphism for the glutathione synthetase enzyme, so some cases of HAGMA with 5-oxoprolinemia occur later in life under particular metabolic stress, suggesting a previously unrecognized genetic defect [5]. Aside from the polymorphism, there also exists acquired glutathione synthetase deficiency because the enzyme may not perform equally well across the person’s life span.

Hereditary 5-oxoprolinemia is a result of autosomal recessive deficiencies caused by hereditary error in one of the enzymes involved in the gamma-glutamyl cycle, but these rare cases usually present in childhood with hemolytic anemia and neurological defects.

In the adult population, a clinically significant acquired transient form of 5-oxoprolinemia may occur, with resultant accumulation of 5-oxoproline and subsequent development of anion gap acidosis. This temporary form of 5-oxoprolinemia may be encountered at any age, and is believed to result from disturbance of the gamma-glutamyl cycle. Risk factors that predispose to glutathione depletion include malnutrition, diabetes, pregnancy and liver disease. Renal impairment also causes reduced urinary excretion of 5-oxoproline. We need to emphasize that our patient demonstrated a number of these risk factors: she was elderly and malnourished, and had diabetes and chronic kidney disease as well as sepsis, and had been unwell for three months.

Several drugs have been implicated in temporary 5-oxoprolinemia. The antibiotics flucloxacillin and netilmicin are thought to inhibit the enzyme 5-oxoprolinase and hence lead to accumulation of 5-oxoproline. Acetaminophen and vigabatrin deplete glutathione stores leading to loss of feedback inhibition and increased production of 5-oxoproline from its precursor gamma glutamylcysteine.

Most of the described cases of transient 5-oxoprolinemia are caused by repeated therapeutic acetaminophen ingestion, usually arising on the background of the aforementioned risk factors [6–8]. The cases of HAGMA with 5-oxoprolinemia after acetaminophen use generally do not have other indicators of acetaminophen toxicity, such as elevated hepatic transaminases or abnormal prothrombin time.

A diagnosis of 5-oxoprolinemia should be considered in any patient with unexplained metabolic acidosis and a history of therapeutic acetaminophen dosing, particularly those patients with coexisting conditions that may decrease the glutathione stores. Patient management should comprise prompt discontinuation of medications that are linked to the development of the condition along with supportive care. Any underlying infection or medical condition must be identified and treated appropriately.

There is no evidence to support that N-acetylcysteine treats or corrects 5-oxoprolinemia. Some studies recommend N-acetylcysteine supplementation to provide the cysteine necessary for glutathione synthesis [9, 10]. However, these studies do not prove that N-acetylcysteine changes the metabolic fate of gamma glutamylcysteine (which already contains cysteine). In patients who are accumulating gamma glutamylcysteine because they lack the last building block for glutathione, despite normal glutathione synthetase activity, glycine may be needed (see Figure 1).

## Conclusion

A diagnosis of 5-oxoprolinemia should be considered in patients with unexplained HAGMA, particularly patients with recent acetaminophen use in combination with sepsis, malnutrition, liver disease, pregnancy or renal failure. Simple urinary tests for organic acids can support the diagnosis of 5-oxoprolinemia. Patient management should include discontinuation of the offending medications, supportive management, and aggressive treatment of the associated risk factors, for example, sepsis. The role of N-acetylcysteine in the management of 5-oxoprolinemia without acetaminophen-induced hepatic injury is unclear.

## Consent

Written informed consent was obtained from the patient for publication of this case report and accompanying images. A copy of the written consent is available for review by the Editor-in-Chief of this journal.

## Abbreviations

HMGA: high anion gap metabolic acidosis.
